# How Is This?

**Published:** 1888-10

**Authors:** 


### HOW IS THIS?

A physician of prominence in Central New York sends us a letter, lately received from a relative. From it we quote:

"We have had recently a homœopathic physician stop with us over night. He left us certain medicines adapted to certain ailments, and asked us to make use of them in our own family, and if successful to recommend them to our neighbors and friends. The doctor also left us a copy of Bœricke & Tafel's *Medical Index,* a key to the treatment of common ailments. Now, Kittie has been troubled with constipation so long that it has almost become chronic, yet she absolutely refuses to take his medicines without first consulting you. You will remember that once upon a time you treated her for a certain ailment with *complete success,* hence her faith in your advice. Now this doctor gave us, as remedies for constipation, 716 Sulphur, taken in the morning, and 501 Nux-vomica, to be taken in the evening; he gave it as his opinion that these would work a complete cure. He also left 28 Aconite, 420 Ipecacuanha, 477 Lycopodium, 603 Phytolacca, 632 Pulsatilla. Do these remedies correspond with your treatment?

"The doctor said he was traveling about from pure love of Homœopathy, and did not expect to get the confidence of the people until by the use of these remedies they became satisfied that his advice was right. He also asked that we study the *Index,* suggesting that by a knowledge of it we might be able to take care of our own family, except in sudden or extreme cases."

We do not know who is responsible for this itinerant physician, but we do believe he is doing much harm both to Homœopathy and to its clients. Those of the laity who use such guides as this *Medical Index* will find that they aggravate simple diseases or maybe render grave cases hopeless. Every homœopathic physician should do his utmost to discountenance all such missionary work. The *Medical Index* referred to in this letter is simply a farce, pure trash; no physician ever knew enough of Homœopathy to use this so called *Index;* how, then, could a layman use it? Here is its advice under head of "FEVERS: Simple, Aconite; aguish, Arsenicum; scarlet, Aconite and Belladonna; rheumatic, Aconite and Rhus-tox."

